# Hierarchical Logic Models as a Tool to Evaluate Programmatic Initiatives: Practical Solutions to Identified Problems

**DOI:** 10.4172/2161-0711.1000522

**Published:** 2017-05-08

**Authors:** S Newbill, A Wickman, C Brown, D Helitzer

**Affiliations:** 1 Director, Folkstone: Evaluation Anthropology and Department of Family and Community Medicine, University of New Mexico, USA; 2 Southwest Center for Agricultural Injury Prevention and Education, USA; 3 Center for Health, Work and Environment, University of Colorado Denver, USA; 4 College of Population Health, University of New Mexico Health Sciences Center, USA

**Keywords:** Hierarchical logic model, Program evaluation, Program planning, Logic model, Institutional grant, Center grant

## Abstract

**Introduction:**

Large programmatic grants advance the missions of funding agencies or organizations. This article describes the programmatic impact of using “hierarchical” logic models in two Centers funded by the National Institute of Occupational Safety and Health (NIOSH) that were designed to achieve NIOSH goals. Such models are supportive of priority setting, policy implementation, and effective evaluation.

**Methods:**

Two NIOSH Centers, an Agricultural Center and an Occupational Safety and Health Education and Research Center, used the same hierarchical logic model process to support the NIOSH programmatic goal of improving worker health and safety in their respective occupational categories. The logic model development processes were led by the same evaluator.

**Results:**

Case studies describe the utilization of “hierarchical” logic models: in each case, NIOSH was the “grandparent”, the Center was its descendant (parent) and the cores were the children. This lineage was articulated through the Center-wide logic model and through the logic model of each of its core programmatic areas (core). The Center-wide logic model ensured that the Center’s goals, and the intended outcomes and impact of its work were linked to the mission and goals of NIOSH. Each core’s logic model articulated how its goals, activities, and outcomes were specifically linked to the Center-wide model.

**Discussion:**

A hierarchical logic model process ensures that the objectives of the funding agency or organization are addressed, and enables stakeholders to articulate the linkages between each layer. This facilitates the process of developing, implementing and evaluating programmatic elements within the framework of strategic planning.

## Introduction

Program planners, evaluators and other stakeholders use logic models to clearly articulate the links between proposed program goals, resources, activities, outcomes, and impacts of their initiatives. This systemic approach to program planning and evaluation provides a road map for the program and assists programs to define strategies that will lead to success. In building logic models, stakeholders must define a program theory to provide a rationale for how the activities will lead to outcomes [1–5]. Involving stakeholders in the development of program logic models instills a shared understanding of the program theory. When working with a large, multi-component program, the integration of logic models can provide further clarification about the program, as a whole, as well as individual program components.

Logic models can provide a productive framework for effective planning and a depiction of the process of change of an intervention. Most often presented as sequenced diagrams or flow charts, logic models demonstrate relationships among the following components: Statement of a problem, various causal and mitigating factors related to that problem, available resources to address the problem, intervention goals and planned activities, and anticipated short and long-term outcomes. This traditional logic model framework may be augmented to include consideration of key factors that may hinder and/or enhance the well-being of the target population, or attainment of the goal set, and may affect the program at the individual, family, programmatic/organizational or policy level.

**Figure 1:**
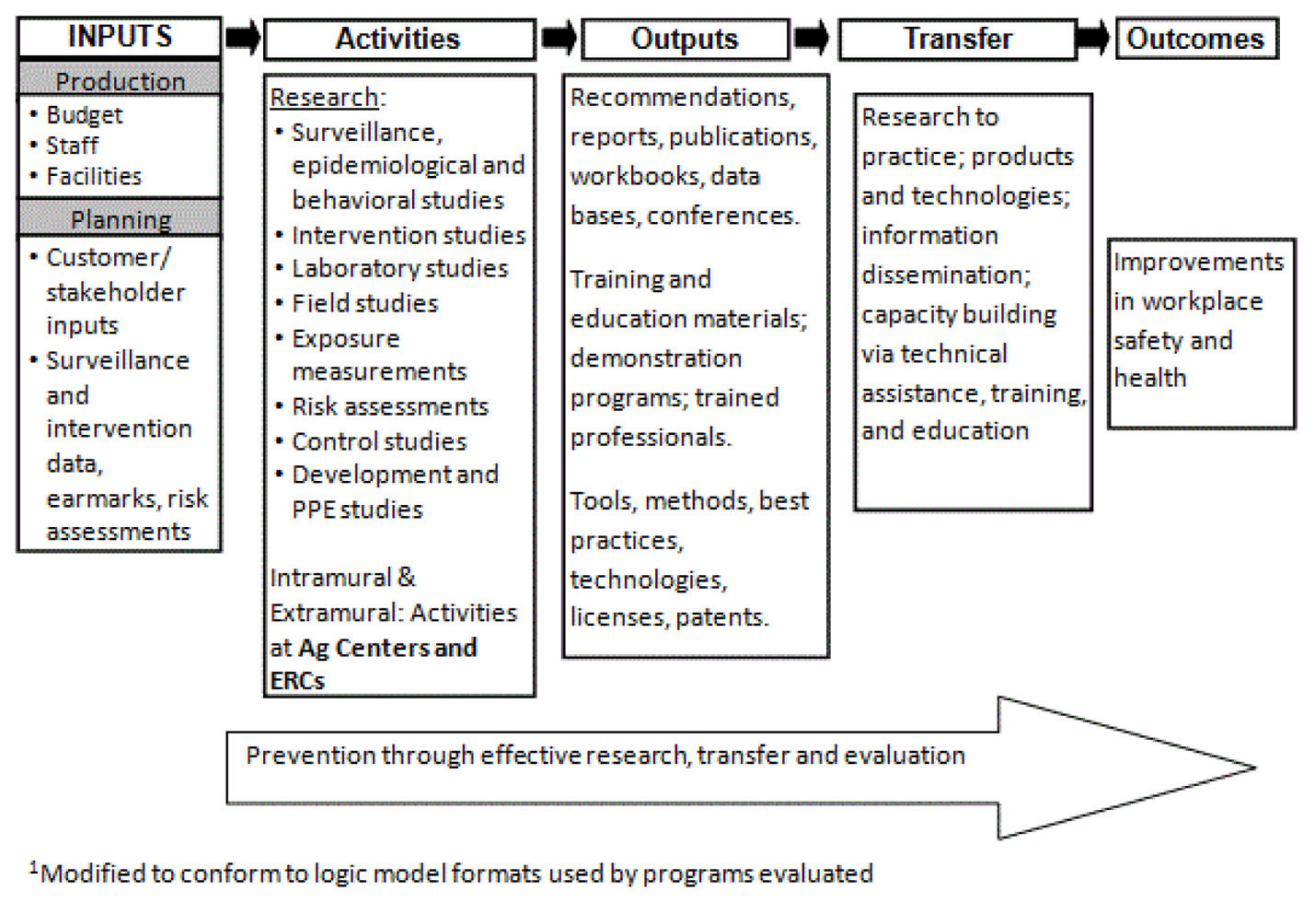
NIOSH logic model.

Institutes and organizations often fund large programmatic grants to support a specific mission or goal. In that case, it is particularly important that the programmatic grant demonstrate the ways in which it will serve to advance that mission. A “hierarchical” logic model process is one way to depict these relationships (Figure 1). The purpose of this article is to describe the hierarchical logic model process and to provide two examples of how this process has been used to support two different programmatic initiatives. These initiatives are the Southwest Center for Agricultural Injury Prevention and Education (SW Center) and the Mountain and Plains Education and Research Center (MAP ERC). Both are funded by the National Institute of Occupational Safety and Health (NIOSH) to meet its goals to improve worker health and safety. The authors, who are evaluators and/or administrators for these two different NIOSH centers, came together to discuss how the hierarchical logic model process gave direction to the framework of the evaluation plans for each Center.

### Program evaluation context

NIOSH funds programs to support occupational safety and health research and education. Their theoretical framework (i.e., program theory) is that in creating infrastructure to support programmatic activities with sets of “core” resources, worker occupational health and safety will be improved and occupational injury and fatalities will be decreased. NISOH has invested resources in 10 Agricultural Safety and Health Research Centers (Ag Centers); 18 regional university-based Education and Research Centers (ERCs); and 34 Training Project Grants that train occupational health professionals and researchers to help meet the increasing demand for occupational physicians, occupational nurses, industrial hygienists and safety professionals (http://www.cdc.gov/niosh/oep/agctrhom.html). The NIOSH-supported Ag/ERC Centers also conduct research and implement prevention projects to address the Nation’s occupational health and safety problems. These programs are all operated out of the Office of the Director and managed by the Director of the Office of Extramural Coordination and Special Projects. As special projects of the Education and Information Division, Training Research and Evaluation Branch, they are vulnerable to budget cuts. Therefore, it is even more important that they demonstrate that they advance the NIOSH mission of improving worker occupational health and safety.

NIOSH research is driven by the National Occupational Research Agenda (NORA), which is organized into 10 programs representing different industry sectors. Since 1996, NORA has become the national research framework for occupational safety and health. NIOSH collaborates with many organizations (e.g., industry, labor, government, academia) to advance occupational health and safety research. The collaboration may be as research partners, users of NIOSH technology and scientific findings, peer reviewers, recipients of research grants and contracts, or sources of equipment, technology, or knowledge for advancing research.

Over time NIOSH guidance for program evaluation and requirements improved. Yet, at the time of this study, the program announcements listed evaluation as one of required administrative components, but did not provide substantive direction as to how the evaluation should be conducted.

### NIOSH Ag center initiative

In order to assist in the NIOSH mission of improving the health of farmers, fishers, and foresters, 10 Ag Centers were established in 1990 as part of the National Program for Occupational Safety and Health in Agriculture. Each Ag Center conducts surveillance, research, education, and prevention projects to address regional agricultural safety and health issues. Each Ag Center fills a unique geographic niche to engage community stakeholders in initiatives to reduce health disparities among disadvantaged groups. A 2012 review of the Ag Center initiatives found their impact to be notable, and highlighted the significant reduction in child death rates due to exposure to agricultural worksite risk and the adoption of safer technologies and/or devices in some agricultural worksites [6].

Each Ag Center re-competes for NIOSH funding every 5 years. In the 2010 proposal application request (PAR), applicants were required to include three major components: Internal cores, research projects and an evaluation program. Internal cores included (1) Administrative and Planning, (2) Feasibility/Pilot Studies and Emerging Issues, and (3) Outreach. Research projects were categorized as Research, Education/Translation or Prevention/Intervention. Each applicant was required to have internal and external advisory committees.

### NIOSH ERC initiative

Established in 1977, the ERCs are part of a network of training grants that ensure an adequate supply of qualified professional occupational safety and health practitioners (OS&H) and researchers. The ERCs, located in each of the 10 Federal Department of Health and Human Services (DHHS) Regions, offer multidisciplinary educational training, continuing education and outreach programs to train OS&H professionals.

Currently, ERCs are housed in 18 academic institutions that address OS&H training and research in a cross-cutting, integrated manner. Each institution provides interdisciplinary graduate training in the core areas of Industrial Hygiene, Occupational Health Nursing, Occupational Medicine, Occupational Safety, and allied OS&H fields. The multidisciplinary approach results in cross-fertilization among the various disciplines. The number of professionals engaged in research and practices to promote occupational safety and health has substantially increased through ERCs initiatives (http://www.cdc.gov/niosh/oep/ercreports.html).

ERCs also conduct continuing education programs for OS&H. They offer training courses for practicing physicians, nurses, industrial hygienists, safety professionals, and other occupational safety and health professionals, paraprofessionals and technicians, including personnel from labor-management health and safety committees. Two essential components of each ERC are outreach and research to practice (R2P) activities that are implemented in collaboration with other institutions, businesses, community groups, or agencies located within the region. ERCs are encouraged to address geographic needs and to implement innovative strategies to impact the practitioner environment.

### Two Cases

The Southwest Center for Agricultural Health, Injury Prevention, and Education (SW Center) was created in late 1995 at the University of Texas Health Northeast to serve Arkansas, Louisiana, New Mexico, Oklahoma and Texas through research and outreach aimed at reducing injuries and fatalities among agriculture, forestry and fishing (AFF) workers and their families. The mission of the SW Center, to improve the safety and health of agricultural, forestry and fishing workers, is accomplished through research, intervention and education projects. These projects build and leverage a network of strategic partners who represent the diversity of the workforce and the range of agricultural production in the region. The SW Center is guided by an External Advisory Committee (EAC) and an Internal Advisory Committee (IAC). The EAC is composed of a multidisciplinary group of experts in dairy, agriculture, forestry, logging, beef cattle, veterinary medicine, migrant farmworkers and commercial fishing. These advisors represent expertise from each of the states served by the SW Center. The IAC provides advice to the SW Center director to support effective management of the Center.

The Mountain and Plains Education and Research Center (MAP ERC) was established in 2007 and includes the University of Colorado, Colorado State University, National Jewish Health, Denver Health and Hospital Authority and the University of New Mexico Health Sciences Center. Spanning from the borders of Canada to Mexico, the MAP ERC helps meet the occupational health education and research needs of Colorado, New Mexico, Arizona, Montana, Wyoming, North Dakota and South Dakota. The MAP ERC is guided by an External Advisory Panel (EAP) and an Internal Executive Committee (IEC). The EAP represents labor, industry, government, academic, and professional organizations. The IEC provides advice to the MAP ERC director to support effective management of its Center.

## Methods

The senior author was the evaluator. She worked with the SW Center and the MAP ERC to create logic models for their Centers, programmatic cores and research projects. In both cases, the mission of NIOSH and the purpose of the Centers in attaining NIOSH goals were considered in the development of the program components. Although there was no requirement to use logic models for program planning and evaluation, the evaluator coordinated the effort to incorporate logic models in the program development and evaluation process. This process helped articulate the connection between the Center’s goals and NIOSH objectives, and ensured that the program components supported the Center goals. As a result, both Centers developed Center-wide logic models to address NIOSH goals, the NORA goals, and the specific activities in the NIOSH logic model (Table 1 and Figure 1). Within each Center, the set of logic models is analogous to a family tree: Center-wide (parent); programmatic cores (children); and research projects (grandchildren).

### SW Center

**Figure 2:**
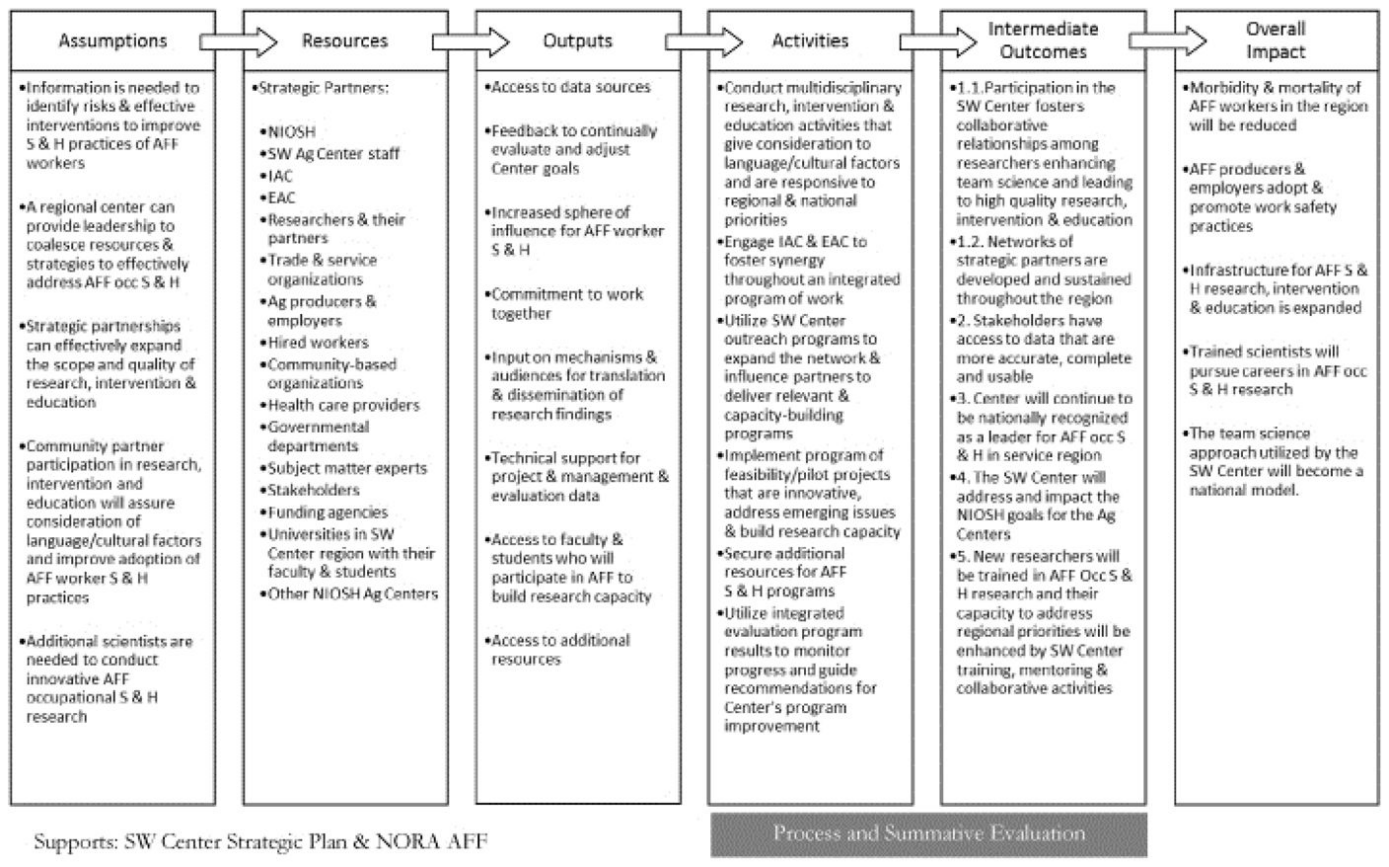
SW Ag Center-wide logic model.

The logic model development process began during the preparation phase for the renewal application for the SW Center in the fall of 2010. The SW Center staff and research PIs worked with the evaluator to create the SW Center-wide logic model and the logic models for the individual components and research projects. Most of the work was done virtually, over the phone and through email. The format used in the SW Center logic models includes six columns: Assumptions, Resources, Outputs, Activities, Intermediate Outcomes, and Overall Impact (Sector and Center) (Figure 2).

The group of hierarchical logic models for the SW Center was: Center-wide (parent), Outreach and Program Evaluation Pilot Studies/Emerging Issues (children), and four Research Projects (grandchildren).

The SW Center staff set out to revise and re-envision its strategic plan at the time the renewal grant was awarded. The logic models were used as resources for this process. A committee was formed from EAC, IAC, PIs, evaluation experts and staff to update the existing SW Center strategic plan. Goals are shown in Table 1.

### MAP ERC

**Figure 3:**
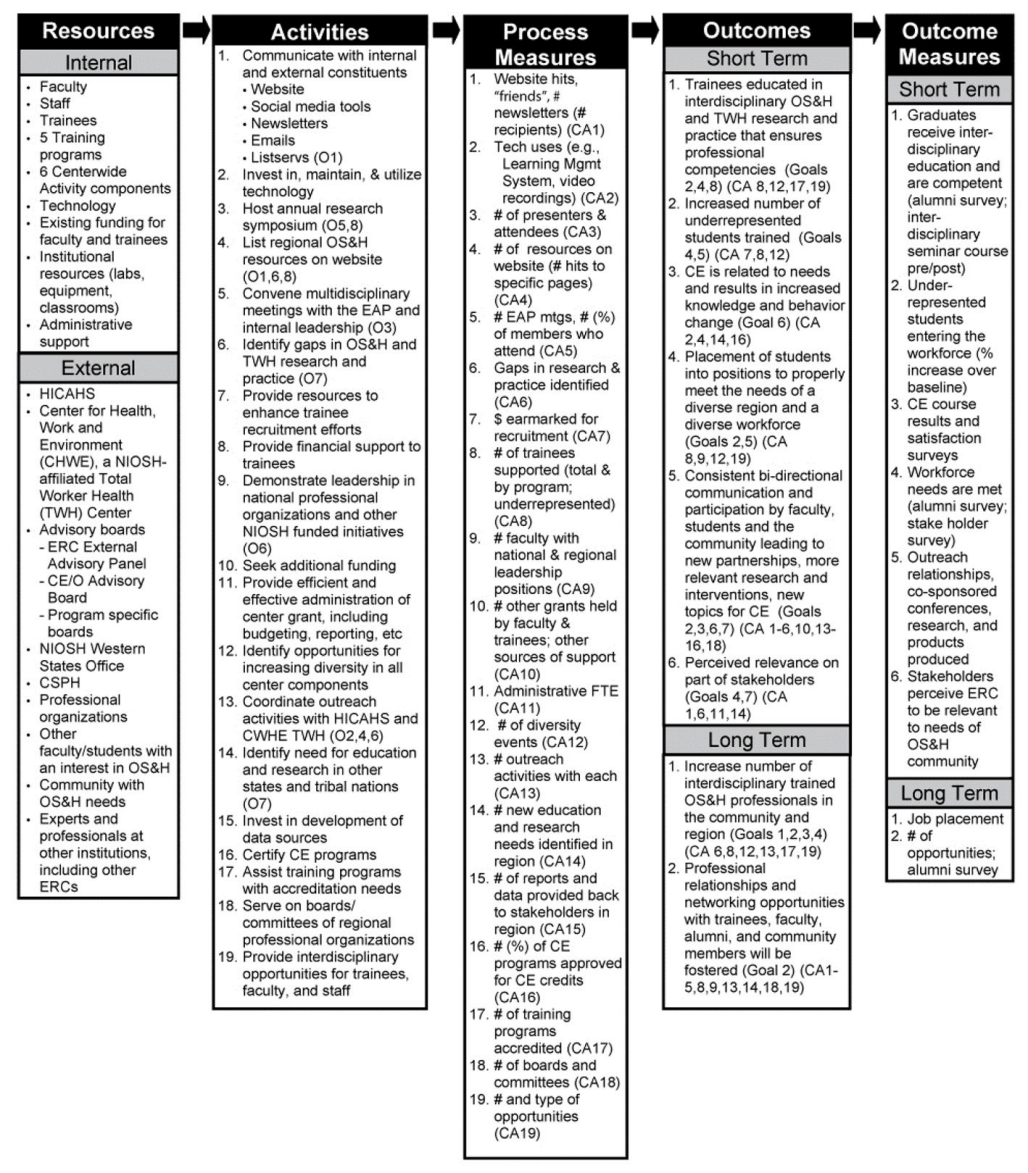
MAP ERC Center-wide logic model.

Strategic planning for the MAP ERC occurred after initial funding and again after the award of the competing renewal in 2010. The MAP ERC goals were developed as part of the application for the Center funding in a process that involved reviewing NIOSH and MAP ERC goals and setting priorities for the funding cycle (Table 1). These priorities are reflected in the MAP ERC Center-wide logic model, in its resources, activities, and short and long term outcomes (Figure 3). The logic model process was a long-term activity, starting after funding was received; the evaluator, the advisory board, ERC staff and faculty convened and spent several days developing the logic models.

The group of logic models for MAP ERC was: Center-Wide (parent), Outreach, Continuing Education, Residency Programs, Interdisciplinary Coordination, Research Training, Community, Diversity, and Program Evaluation (all children). As compared to the set of logic models for the SW Center, the MAP-ERC suite of logic models represents programmatic initiatives; thus there were no grandchildren.

## Results

For this paper, we highlight the hierarchical thread (the family tree) from the NIOSH goal to its corresponding Center goals. As described above, NIOSH was the “grandparent”, the Center was its descendant (parent) and the cores were the children. To demonstrate the hierarchy, each case study below demonstrates the links between one of the Center goals to the goals, objectives, and activities of its Outreach Core, as this component was a requirement for both the SW Center and MAP ERC. In the case of the SW Center, we include the linkages between the Outreach Core and a research project to complete our example. In the ERC, we depict the relationships between the Center and its Outreach and Research Training Cores.

### Case study 1-SW Center

The SW Center Outreach Core engages and expands its network of strategic partners to design, deliver and evaluate educational products and programs to raise awareness of safety and health issues, diminish exposure risks, improve the adoption of best practices, and consequently reduce injuries and fatalities to AFF workers and their families. This is accomplished through regular communication with stakeholders, capacity building activities, and topic/population based interventions that are informed by regional experts. The Outreach Core has its own goals.

The Outreach Core is designed to help the SW Center meet NIOSH Strategic Goals 2 and 3 (Table 1). The NIOSH AFF research logic model describes outputs, including publications, workshops, and conferences, that are the responsibility of the Outreach Core. Transfer from research to practice (R2P) is evidenced by products, technologies, information, capacity building, and training conducted by the SW Center. In this case study, we use the Vietnamese Shrimper research project as the grandchild that provides resources to the Outreach Core so it can accomplish its goals.

During the logic model development process, the Outreach Core aligned its activities to the SW Center-wide Strategic Plan. The items in the activities, outcomes and impact columns from the Outreach Core logic model align with those in the SW Center-wide logic model outcomes and impacts in Figure 2. Most specifically, the overall impact of the Outreach Core and its intermediate outcomes were supportive of SW Center Strategic Goals. Each of the impact and intermediate outcome indicators were aligned with at least one of the SW Center Strategic Goals, and most met more than one goal.

**Figure 4:**
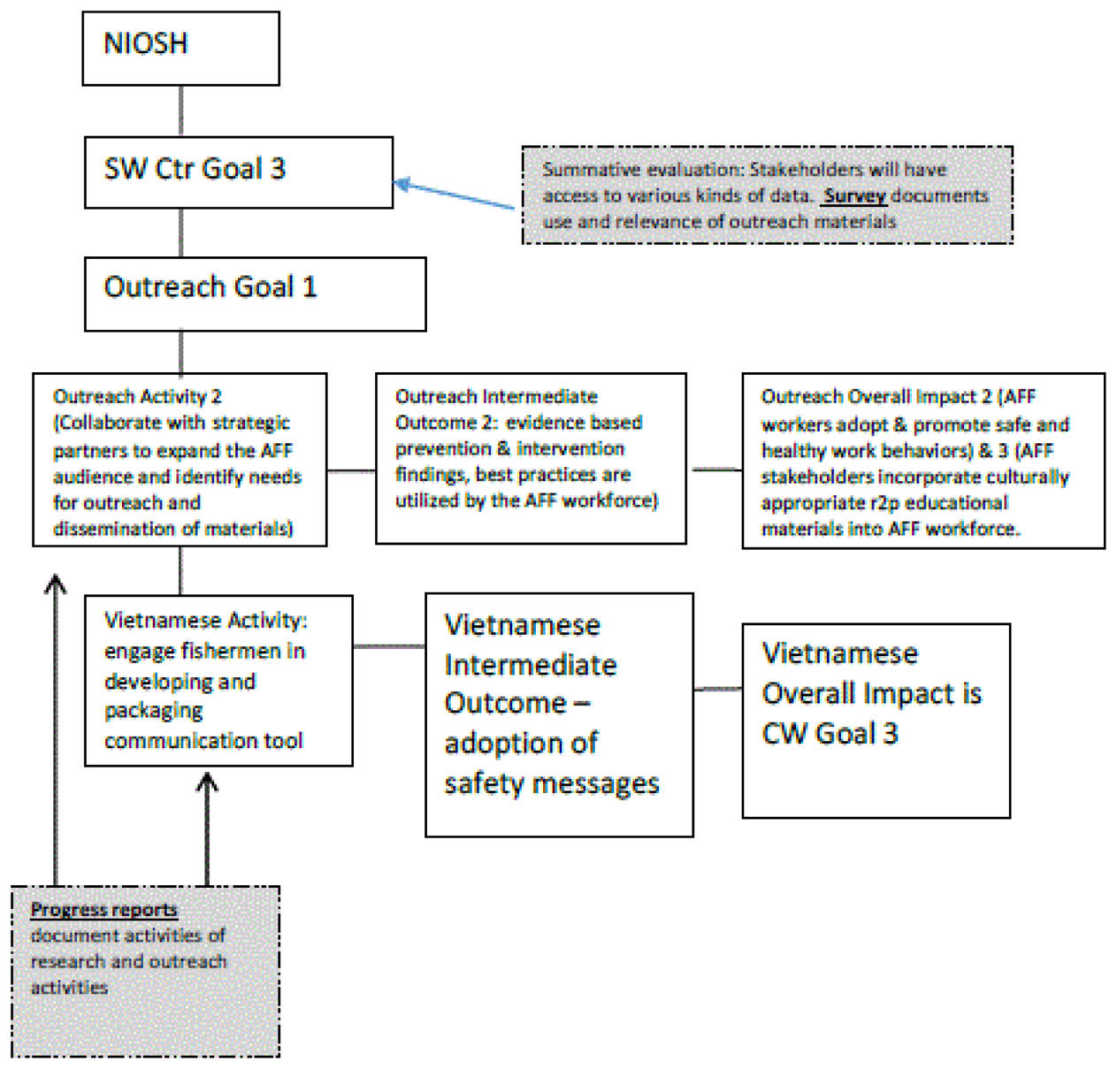
SW Ag Center hierarchy of goals, activities, outcomes, impact and evaluation.

The Outreach Core itself had goals (Table 1), which became the foundation of the Outreach activities. As an example of the linkages (Figure 4), Outreach Core activities were linked to Outreach goals, and those goals were linked to SW Center-wide goals. Further, outreach activities, intermediate outcomes, and impact required the results of the Vietnamese Shrimper project’s activities. Implementation of the Outreach activities was dependent on each of the research projects (in this example, the Vietnamese Shrimper research project) achieving their activities, outcomes and impact.

Similarly, the evaluation followed a hierarchical pathway. Progress reports collected information about the activities in the Vietnamese Shrimper research project and the Outreach Core. The summative evaluation examined the attainment of the Center-Wide goals, the evidence for which are the data documenting activities, intermediate outcomes and impact of the Outreach Core and the Vietnamese Shrimper research project.

### Case study 2-MAP ERC

One role of the MAP ERC Outreach Core was to convene the community OS&H workforce in the region, in collaboration with professional societies, labor organizations, Native American and Latino communities, government, industry groups, community organizations, and other ERCs. Hundreds of organizations were engaged in raising the profile of OS&H issues among the broader community.

**Figure 5:**
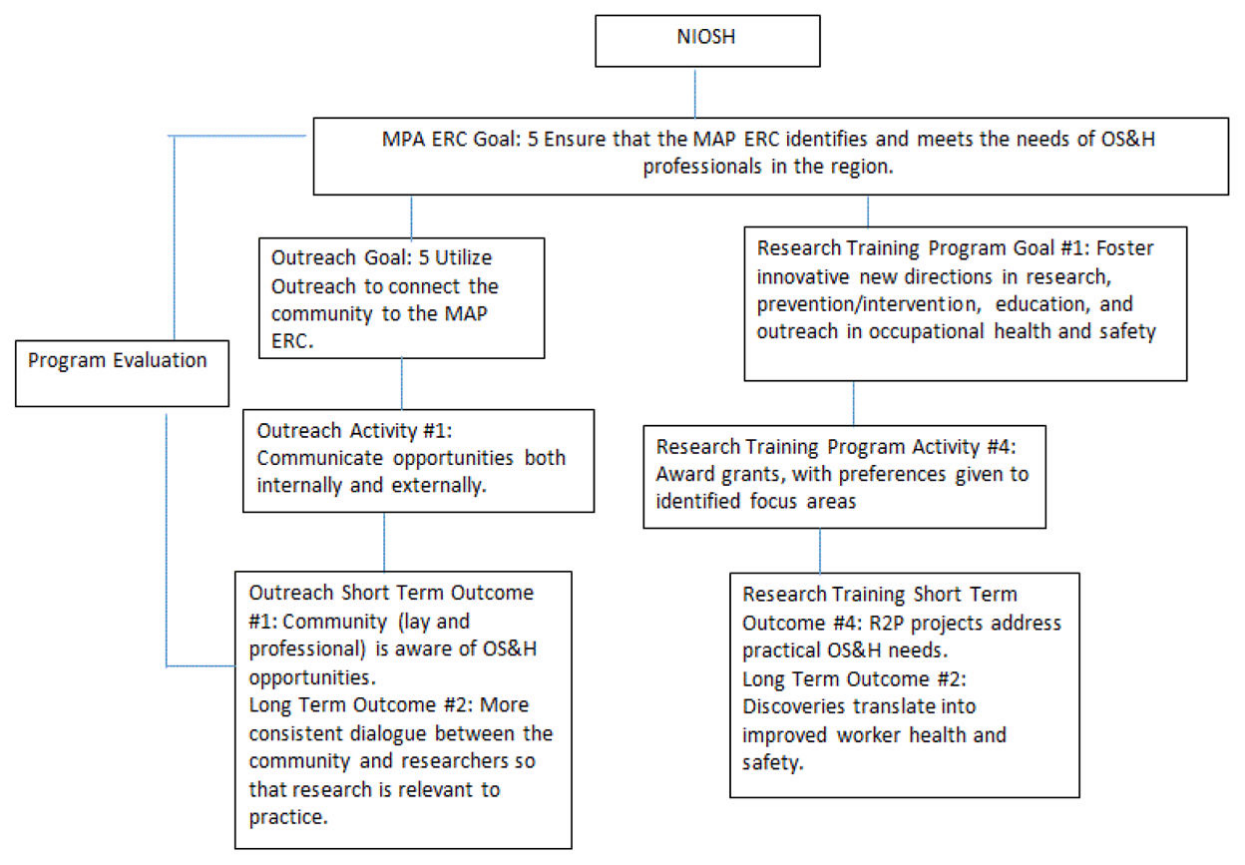
MAP ERC hierarchy of goals, activities, outcomes, impact and evaluation.

The MAP ERC Outreach Core logic model provides detail about the relationship between the MAP ERC Center-wide goals and the goals and outcomes for the Outreach Core. Similar to the SW Center, the Outreach Core created its own goals (Table 1). Within the Outreach Core logic model, the activities are linked to process measures and to short- and long-term outcomes. Different from the SW Center, the MAP ERC Center-wide goals were met by multiple programmatic cores. One example is the Center-wide Goal 5-met by the Outreach Goals 2 and 5. The Center-wide goal was also met by the Research Training Core Goal 1 (Figure 5).

## Discussion and Conclusions

The case studies described above demonstrate the ways in which a hierarchical logic model process may be used to link goals and outcomes between the funder (i.e., NIOSH), and two of its major initiatives, the Ag Centers and the ERCs. NIOSH articulated its goals for the Ag Centers and the ERCs (Figure 1) in its logic model. As is described in this manuscript, the NIOSH logic model is used as the “grandparent” for those of its extramural programs, such as the Ag Center Initiative and the Education and Research Center initiatives, both “parents”. In the SW Center, the Outreach Core (child) supported the Center, and the research projects (grandchildren) supported the Outreach Core. In the MAP ERC, the Outreach and the Research Training Cores (children) supported the Center.

The senior author worked as the leader of the evaluation for both the SW Center and the MAP ERC. In both cases, the logic model process began in the application period and was refined once the Center was funded. The SW Center was funded in September 2012 and the MAP ERC was funded in August 2010. The results described in this manuscript depict evaluation in action: each set of hierarchies was tailored to the structure and lineage of each Center and what was needed to accomplish each Center’s goals, which were determined by the NIOSH goals. The major difference between the two hierarchies is that one favors depth (SW Center) and the other favor breadth (MAP ERC).

The logic model hierarchy mirrors the multi-level and nested characteristics that underlie the rationale for hierarchical linear modeling in statistics [7] and the parent-child relationships that the form foundation of content analysis software programs such as QSR NVivo and Atlas Ti.

Hierarchical logic models are appropriate for all large intramural and extramural program projects, because they reflect the goals of the agency (in this case NIOSH) and therefore the rationale for the program project or initiative. The hierarchical logic model process helps agencies prepare funding announcements and reports to Congress or other constituencies that assess the degree to which an agency is attaining its own goals. As an example, the evaluation of the Ag Centers by the National Academies of Science was facilitated by explicit linkages between agency goals, objectives and initiatives and the corresponding goals, objectives and activities of the Centers [6].

The hierarchical logic model approach is very helpful at the Center level for orientation of all stakeholders to include internal and external advisory boards, site monitors and study sections to understand program goals, practices, and rationale. Furthermore, the hierarchical logic model process offers a common purpose between the evaluation team, program staff, and stakeholders.

The hierarchical logic model approach allowed the MAP ERC personnel to further prioritize areas where multiple programmatic areas were designed to meet the same goals and coordinate activities, tracking and increased impact. This coordination of activities minimizes duplication of efforts and central tracking allows for higher level decisions about what activities are more successful than others, across programs.

The logic models are a living portrait of evaluation in action. The use of a hierarchical logic model process aids the federal government to manage, integrate and coordinate a large complex system of varied and diverse programmatic elements, thereby ensuring that its goals are attained.

## Figures and Tables

**Table 1:** Goals of NIOSH, SW Center and MAP ERC during evaluation period, 2011–2015.

**NIOSH Goals**
Conduct research to reduce work-related illnesses and injuries
Promote safe and healthy workplaces through interventions, recommendations and capacity building
Enhance international workplace safety and health through global collaborations
**SW Center Goals**
Identify and characterize ongoing and emerging issues within AFF occupational safety and health
Translate AFF occupational safety and health basic and applied research into practice
Increase awareness and visibility of AFF occupational safety and health
Expand financial and human resources to sustain and grow the mission of the Center
**SW Center Outreach Core Goals**
Develop a structured communication network of partners to identify regional safety and health needs and to disseminate prevention/intervention findings, best practices, tools, approaches, technologies, guidelines, and policies
Enhance the capacity of regional agricultural educators, producers, and stakeholders as well as community competence to sustain SW Center-initiated outreach projects
Identify outreach and education interventions through topic/population based initiatives that will serve as models for the promotion of safe and healthy work behaviors
Increase awareness of AFF safety and health careers among students, current researchers, educators and social scientists
**MAP ERC Goals**
Create and maintain a framework for supporting training, research, and continuing education for OS&H that promotes diversity, cultural sensitivity, leadership development, and interdisciplinary collaboration to meet the needs of the region
Enhance interdisciplinary and inter-institutional education and research in OS&H, including Total Worker Health (TWH)
Ensure that MAP ERC supported research and demonstration projects address regional needs and identified risks, and contribute to improvements in worker health and wellbeing
Ensure that all center components incorporate efforts to enhance representation and engagement with diverse and vulnerable populations (diversity)
Ensure that the MAP ERC identifies and meets the needs of OS&H professionals in the region
Ensure the sustainability of the MAP ERC by demonstrating leadership and collaboration with national and regional stakeholders, funding agencies, and alumni
Ensure that data sources, including surveillance data, better document trends and opportunities for intervention in the region
Ensure that the components of the Center meet standards of practice (including accreditations)
**MAP ERC outreach core goals**
Build upon existing OS&H resources and promote and document their use
Increase awareness of OS&H issues and professions in the community
Develop and target outreach activities to worker populations who are underserved
Increase appreciation of outreach and dissemination to the community as a scientific endeavor to enhancing health promotion and disease prevention
Utilize outreach to connect the community to the MAP ERC and help the MAP ERC be aware of community needs
